# The health of children and adolescents with a migration background in Germany – Results of the cross-sectional KiGGS Wave 2 study

**DOI:** 10.25646/6074

**Published:** 2019-09-18

**Authors:** Carmen Koschollek, Susanne Bartig, Alexander Rommel, Claudia Santos-Hövener, Thomas Lampert

**Affiliations:** Robert Koch Institute, Berlin Department of Epidemiology and Health Monitoring

**Keywords:** MIGRATION, MIGRATION BACKGROUND, KIGGS, HEALTH MONITORING, HEALTH REPORTING

## Abstract

Over a third (36.5%) of young people living in Germany have a migration background. Based on the data of the second follow-up to the German Health Interview and Examination Survey for Children and Adolescents (KiGGS Wave 2, 2014-2017), the health situation of 11- to 17-year-olds with and without a migration background is described using selected indicators. In order to account for the diversity among children and adolescents with a migration background, the health indicators used in this study are stratified by migration background but also by additional migration-related characteristics. In addition, the results from the various subgroups are also stratified by sociodemographic characteristics. No differences in self-assessed general health or the outpatient utilisation of paediatric and general medical services were identified between 11- to 17-year-olds with and without a migration background. However, migration-related differences were identified in health behaviour: whereas children and adolescents with a one- or two-sided migration background are more frequently overweight (including obesity), they consume risky amounts of alcohol less often than those of the same age without a migration background. Finally, the outcomes of the indicators also vary according to migration-related and sociodemographic characteristics.

## 1. Introduction

Data from the 2017 microcensus demonstrate that around 19.3 million people in Germany have a migration background. As such, almost a quarter of the population in Germany (23.6%) was born either themselves or at least one parent without German citizenship. More than one third of the young people in Germany (36.5%) have a migration background and 20.3% of the migrant population under the age of 18 have experienced migration themselves; in other words, they were born abroad and moved to Germany at a later date [1]. International migration can be defined as the cross-border relocation of a person’s permanent place of residence [2, 3]. This biographical event can influence the health situation of migrants and their children born in Germany (second-generation migrant) through various factors before, during and after migration [4, 5]. Although children and adolescents born in Germany are neither directly affected by the living conditions found in their parents’ country of origin nor by the process of migration itself, the situation can still influence their health (for example, due to discrimination). At the same time, the socialisation of children and adolescents with a migration background may be marked by cultural conflicts: in addition to their own family’s values and ways of life, children with a migration background are also influenced by the ways of thinking and the norms characterising the host society. On the other hand, children and adolescents can also influence the health of their parents by mediating during their parents’ interactions with health care institutions [5-7].

**KiGGS Wave 2**

Second follow-up to the German Health Interview and Examination Survey for Children and Adolescents

**Data owner:** Robert Koch Institute

**Aim:** Providing reliable information on health status, health-related behaviour, living conditions, protective and risk factors, and health care among children, adolescents and young adults living in Germany, with the possibility of trend and longitudinal analyses

**Study design**: Combined cross-sectional and cohort study

**Cross-sectional study in KiGGS Wave 2**

**Age range:** 0-17 years

**Population:** Children and adolescents with permanent residence in Germany

**Sampling:** Samples from official residency registries - randomly selected children and adolescents from the 167 cities and municipalities covered by the KiGGS baseline study

**Sample size:** 15,023 participants

**KiGGS cohort study in KiGGS Wave 2**

**Age range:** 10-31 years

**Sampling:** Re-invitation of everyone who took part in the KiGGS baseline study and who was willing to participate in a follow-up

**Sample size:** 10,853 participants

**KiGGS survey waves**

▶ KiGGS baseline study (2003-2006), examination and interview survey
▶ KiGGS Wave 1 (2009-2012), interview survey
▶ KiGGS Wave 2 (2014-2017), examination and interview survey

More information is available at www.kiggs-studie.de/english

Migration status alone is an inadequate means of analysing health-related inequalities [8, 9]. As such, further explanatory factors, such as a person’s social situation, also need to be taken into account when studying health inequalities [10]. However, the data available to describe the health of the population with a migration background is inadequate. For example, the proportion of migrants in health surveys often does not correspond to their proportion of the population. In addition to peculiarities in participant behaviour, linguistic barriers, mistrust and concerns about sensitive questions or consequences concerning the residence status of (non-)participation lead to lower rates of survey participation among people with a migration background [11, 12]. Therefore, a migration-sensitive approach is essential in order to better involve people with a migration background in health surveys.

The German Health Interview and Examination Survey for Children and Adolescent (KiGGS) regularly collects nationwide representative data on the health of children and adolescents. The second follow-up survey (KiGGS Wave 2, 2014-2017), which was designed as an interview and examination survey, implemented a multi-level migration-sensitive approach with the aim of increasing the participation of families with a migration background. In addition to an oversampling of children and adolescents without a German citizenship, an onomastic procedure was used to send out invitations to prospective participants in the language of their probable country of origin. Additionally, the survey materials were translated and the field teams received intercultural training [13].The results from the KiGGS baseline study (2003-2006), which was the first to implement a migration-sensitive study design as part of the Robert Koch Institute’s health monitoring framework, indicate differences in the health situation between children and adolescents with and without a migration background. The study found that a significantly lower proportion of 11- to 17-year-olds with a two-sided migration background rate their health as good or very good compared to those without or with a one-sided migration background. In addition, children and adolescents with a two-sided migration background are disproportionately affected by overweight and obesity. However, they also consume alcohol less frequently than the comparison groups (children and adolescents with a one-sided or without a migration background). Overall, the results from the KiGGS baseline study indicate a high level of heterogeneity among children and adolescents with a migration background. As such, individual health indicators may vary considerably by sex, country of origin and the length of stay [14]. In this regard, it is essential that studies go beyond merely distinguishing between the presence or lack of a migration background.

Based on the set of core indicators of the Federal Health Reporting on people with a migration background, the health situation of 11- to 17-year-old children and adolescents with and without a migration background is described using selected health indicators collected in KiGGS Wave 2 (see also the article on Health reporting on people with migration background – Selection and definition of (core) indicators in this issue of the Journal of Health Monitoring). In line with the recommendations within the referred paper, the results are stratified by migration-related and sociodemographic characteristics.

## 2. Methodology

### 2.1 Study design

KiGGS is part of the health monitoring system at the Robert Koch Institute and includes repeated cross-sectional surveys of children and adolescents aged 0 to 17 (KiGGS cross-sectional study) that are representative for Germany. The KiGGS baseline study (2003-2006) was conducted as an examination and interview survey, the first follow-up study (KiGGS Wave 1, 2009-2012) as a telephone-based interview survey and KiGGS Wave 2 (2014-2017) as an examination and interview survey. The concept and design of KiGGS have been described in detail elsewhere [15-18].

Prospective participants were randomly selected for KiGGS Wave 2 from the official residency registries of the 167 representative cities and municipalities that were chosen for the baseline study. A number of measures have been conducted to increase the study participation and to ensure that the sample was representative of the population in Germany [13, 18].

Overall, 15,023 children and adolescents (7,538 girls, 7,485 boys) took part in KiGGS Wave 2 (response rate 40.1%). A total of 3,567 children and adolescents (1,801 girls, 1,766 boys) participated in the examinations (response rate 41.5%). The response rate was calculated in accordance with the American Association for Public Opinion Research’s (AAPOR) Response Rate 2 [19].

### 2.2 Indicators

This article describes four health indicators from different fields of action from the core indicator set on the health of people with a migration background of the Federal Health Reporting. In addition to self-assessed general health (field of action: promoting and strengthening health), prevalence of overweight (including obesity) and risky alcohol consumption (field of action: promoting and strengthening health-conscious behaviour), this article also describes the utilisation of outpatient medical care provided by general practitioners and paediatricians (field of action: promotion of equal access to health care services). The results are stratified by migration background (without migration background versus one-sided or two-sided migration background), by migration-related characteristics (parental length of stay in Germany and language spoken at home) and sociodemographic characteristics (sex, age and socioeconomic status).

The analyses are based on self-reported information provided by children and adolescents aged between 11 and 17 and – for some indicators – on information provided by their parents. Participants aged 11 to 17 were only considered if they and their parents had provided a valid questionnaire (n=6,103; 3,195 girls and 2,908 boys). Missing values resulted in differing numbers of participants for individual indicators

#### Health indicators

##### General health

This study uses self-assessed general health to describe the health of children and adolescents with and without a migration background. The results on subjective health are based on self-assessments provided by the 11- to 17-year-olds. In accordance with the recommendations of the World Health Organization (WHO) [20], young people were asked ‘How is your health in general?’ Response categories were ‘very good’, ‘good’, ‘average’, ‘poor’ and ‘very poor’. In the following the proportion of children and adolescents who assessed their overall health as ‘good’ or ‘very good’ is reported. As such, the analyses are based on data from 4,937 children and adolescents aged between 11 and 17 without a migration background (2,552 girls, 2,385 boys) and 1,093 peers with a migration background (604 girls, 489 boys).

##### Body Mass Index

The body mass index (BMI), a measurement of a person’s body weight divided by the square of body height (kg/m^2^), is used to classify underweight, normal weight, overweight and obesity. Due to growth-related changes in childhood and adolescence, the BMI values are divided into percentiles that account for differences in age and sex [21]. In Germany, the reference system of Kromeyer-Hauschild is used to define the BMI classifications. Children and adolescents with a BMI above the 90th percentile are classified as overweight, and those with a BMI above the 97th percentile are classified as obese [22, 23]. This core indicator describes the proportion of children and adolescents affected by overweight, including obesity. The data on body weight and height used in the analyses were collected directly from the children aged 11 or above by self-assessment. The analyses include valid data from 4,739 children and adolescents without a migration background (2,447 girls, 2,292 boys) and from 1,022 peers with a migration background (563 girls, 459 boys).

##### Alcohol consumption

The KiGGS study collected data on the lifetime prevalence of alcohol consumption among children and adolescents aged 11 or above. The participants were asked ‘Have you ever drunk alcohol?’ and could answer either ‘Yes’ or ‘No’. The prevalence of risky alcohol consumption was assessed using the Alcohol Use Disorders Identification Test (-Consumption, AUDIT-C), a short test used to measure the level and frequency of alcohol consumption [24]. First of all, the frequency of alcohol consumption is assessed by asking the participants the following question: ‘How often do you have a drink containing alcohol, such as a glass of wine, beer, mixers, spirits or liqueurs?’ The following response categories were provided: ‘Never’, ‘Monthly or less’, ‘2 to 4 times a month’, ‘2 to 3 times a week’ and ‘4 or more times a week’. Data was then collected on the number of alcoholic drinks that were typically consumed in one day (the following answer categories were available: ‘1 to 2’, ‘3 to 4’, ‘5 to 6’, ‘7 to 9’, ‘10 or more alcoholic drinks’). A short additional note served to ensure that responses remained objective: ‘A drink refers to a small bottle of beer = 0.33 l, a small glass of wine or champagne = 0.125 l, a double spirit or liqueur = 4 cl, or a mixer drink = 1 Alcopop’). AUDIT-C’s third question is about heavy episodic drinking, which is defined as the consumption of six or more alcoholic drinks on one occasion at least once a month (response categories: ‘Never’, ‘Less than monthly’, ‘Monthly’, ‘Weekly’, ‘Daily or almost daily’). A total score was calculated from the answers provided to these three questions in order to depict the distribution of risky alcohol consumption. Valid data for risky alcohol consumption are available for 4,815 children and adolescents without a migration background (2,504 girls, 2,311 boys) and 1,080 peers with a migration background (594 girls, 486 boys).

##### Utilisation of outpatient medical care

Data on the utilisation of paediatric and general medical services was collected using the following question: ‘Please tell us which doctors in private practice of the following disciplines you have consulted for your child in the last 12 months and how often’ (data provided by the parents of children and adolescents aged between 11 and 13, and by young people themselves aged 14 or above). Explicitly included are ‘home visits, but not doctor contacts in the hospital or in a medical spa’. The response categories ‘Paediatrician’ and ‘General practitioner’ were merged to form a single category. The analyses describe the proportion of children and adolescents who have visited paediatricians and/or general practitioners in the last twelve months. The results are based on valid data from 4,882 children and adolescents without a migration background (2,547 girls, 2,335 boys) and from 1,067 peers with a migration background (592 girls, 475 boys).

#### Characteristics of stratification

##### Sociodemographic characteristics

Prevalences are presented separately for girls and boys. In line with the categories of the German Youth Protection Act, the participants are classified into children (aged between 11 and 13) and adolescents (aged between 14 and 17). A family’s socioeconomic status (SES) was measured through an index based on the information parents provided on educational background, occupational status and income situation (equivalised disposable income) [25]. This enabled the participants to be categorised as either belonging to a family with a low, medium or high SES.

##### Migration-related characteristics

In addition to sex, age and SES, results are stratified by migration background. The definition of the migration background in KiGGS is based both on the country of birth of the child and the parents as well as on the citizenship of the parents. Thus, there is a two-sided migration background if both parents were not born in Germany or do not have the German citizenship. If the child and at least one of its parents was born abroad this is also defined as a two-sided migration background. A one-sided migration background is given if one parent was born abroad or does not have German citizenship [6, 14].

The results are also stratified by a second migration-related characteristic – the length of stay of the child’s parents in Germany. Parents were asked the question ‘Since when has Germany been your main country of residence?’ by either stating ‘Since I was born’ or by providing the year that they came to Germany. The length of stay of parents in Germany was calculated as the difference between the survey year and the year that the parents moved to Germany. Answers provided by the mother were taken into account if she or both parents had moved to Germany; answers from the father were used if he was the only one having moved to Germany. Following categories were built according to the answers: ‘Always or for more than 20 years’, ‘11 to 20 years’ and ‘0 to 10 years’. Thus, the first category also includes children and adolescents that do not have a migration background. Prevalences were also stratified by a third migration-related characteristic – the language spoken at home. Parents were asked ‘Which languages do you speak at home?’ They could choose between ‘German’ or ‘Further languages’. If the parents selected the latter, they could indicate two ‘Further languages’. According to parents’ answers, the categories ‘Only German’, ‘German and further language(s)’ or ‘Only further language(s)’ were built to describe the language spoken at children and adolescents’ homes.

### 2.3 Statistical analysis

The calculations were carried out using a weighting factor that corrects deviations within the sample from the population structure with regard to regional structure (rural area/urban area), age (in years), sex, federal state (as of 31 December 2015), German citizenship (as of 31 December 2014) and the parents’ level of education according to the CASMIN classification (Comparative Analysis of Social Mobility in Industrial Nations [26], Microcensus 2013 [27]). The proportions described in this article represent weighted proportions and may differ accordingly from the reported unweighted case numbers (n). All analyses were performed with Stata 15.1 (Stata Corp., College Station, TX, USA, 2017) using a data set of KiGGS Wave 2 specially compiled for migration-related analyses. Stata survey commands were used during all of the analyses to account for any clustering that might have occurred due to the selection of the sample points and the weighting applied to calculate confidence intervals and p-values [28].The results are presented as prevalences with 95% confidence intervals (95% CI). Prevalences are estimates, the precision of which can be assessed through the use of confidence intervals; wide confidence intervals thereby indicate a greater statistical uncertainty in the results. Univariate logistic regression was used to calculate odds ratios (OR) and p-values in order to measure the strength of a statistical relationship. A statistically significant difference between groups is assumed when the corresponding p-value is smaller than 0.05. Odds ratios indicate the factor by which the statistical chance of a health outcome occurring in a particular group is higher or lower than in the reference group.

## 3. Results

###  

####  

##### The participants’ sociodemographic and migration-related characteristics

Of the 6,103 participants (3,195 girls and 2,908 boys), more than half are aged between 14 and 17 years old (59.4%); slightly less than half are aged between 11 and 13 years (40.6%). Based on data from 6,082 children and adolescents, the majority belongs to the medium SES group (62.0%); around one in five belongs to the low SES group (20.2%) and slightly fewer belong to the high SES group (17.8%).

Three out of four participants do not have a migration background (74.5%); nearly one tenth has a one-sided migration background (9.1%) and approximately one sixth has a two-sided migration background (16.5%). Of the latter, 24.1% have their own experience of migration, and, therefore, moved to Germany from abroad. The most common countries of birth among 11- to 17-year-olds with experiences of migration are Poland (n=17), Syria (n=13) and Russia (n=11). The analyses of parental length of stay are based on data from 5,767 participants. The vast majority of parents have either been living in Germany for more than 20 years or have always lived in the country (88.8%). From 8.1% of participants parents have lived between 11 and 20 years in Germany; only a small minority of parents have lived in Germany for ten years or less (3.1%). Data are available from 6,021 participants on the language spoken in their home. Four out of five children and adolescents speak only German at home (80.2%); about one in six speak German and one or more further languages at home (16.7%). Only a very small minority does not speak German at all at home (3.1%).

##### General health

The vast majority of participants assessed their general health as very good or good. No significant differences were identified between the groups according to migration background (without migration background 89.4%, one-sided migration background 89.1%, two-sided migration background 87.1%). Even further stratification by sex, age and SES, revealed no significant differences in self-assessed general health based on migration background (Annex Table 1).For the other migration-related characteristics there are few significant differences in self-assessed general health. The subgroup analysis demonstrates that girls whose parents have lived in Germany between 11 and 20 years rate their health as very good or good less frequently than girls whose parents have been living in Germany for more than 20 years or who have always lived in the country. The same applies to the 14- to 17-year-old subgroup. Additionally in the group with a low SES, children and adolescents whose parents have lived between 11 and 20 years in Germany rate their own health as very good or good slightly less frequently than those whose parents have lived in Germany for more than 20 years or who have always lived in the country (Annex Table 1).

In general, no significant differences were identified between the participants according to the language spoken at home. However, a significant difference was identified among the boys: boys who speak only German at home rate their health as very good or good less frequently than boys who do not speak German at home. Among 14- to 17-year-olds, those who speak German in addition to one or more further languages at home rate their overall health as very good or good somewhat less frequently than those whose only home language is German (Annex Table 1).

##### Body Mass Index

**Figure 1:**
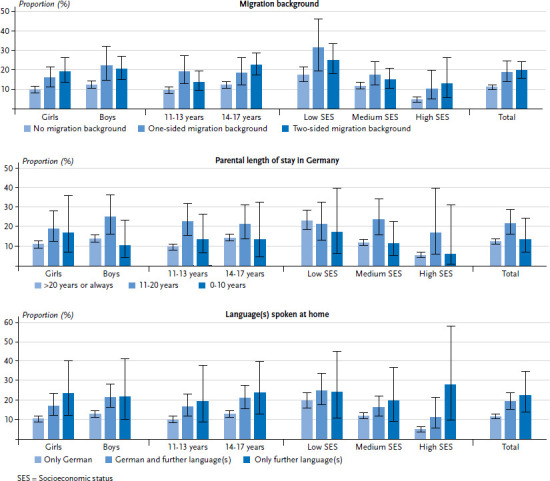
Prevalence of overweight (including obesity) among 11- to 17-year-olds by sociodemographic and migration-related characteristics (n=3,010 girls, n=2,751 boys) Source: KiGGS Wave 2 (2014-2017)

Children and adolescents with a one-sided or two-sided migration background are affected by overweight (including obesity) significantly more frequently than children and adolescents without a migration background (Figure 1 and Annex Table 2). This applies to girls as well as boys. Differences in the frequency of overweight were also identified according to age: whereas 11- to 13-year-old children with a one-sided migration background are more frequently affected by overweight than children without a migration background of the same age, in the group of the 14- to 17-year-olds, overweight is more common among those with a two-sided migration background. Significant differences are also evident in all three SES groups. In the groups with a low or medium SES, 11- to 17-year-olds with a one-sided migration background are more frequently affected by overweight than those without a migration background. In the group with a high SES, young people with a two-sided migration background are more frequently affected by overweight than their peers without a migration background (Figure 1 and Annex Table 2).

Differences were also identified with regard to parental length of stay in Germany. Children and adolescents whose parents have lived in Germany between 11 and 20 years are more frequently affected by overweight than those whose parents have lived in Germany for more than 20 years or for their entire life. This difference is evident among girls and boys. Among 11- to 13-year-olds whose parents have lived in Germany between 11 and 20 years are also more frequently affected by overweight than those whose parents have lived in Germany for more than 20 years or for their entire life. This difference is also found in the groups with a medium or high SES (Figure 1 and Annex Table 2).

A number of differences were also identified in terms of the language spoken at home. Overall, children and adolescents who either speak German at home in addition to one or more further languages or who do not speak German at home are more frequently affected by overweight compared with those who speak only German at home (Figure 1 and Annex Table 2). Both girls and boys who speak one or more further languages in addition to German at home are more frequently affected by overweight than girls and boys whose only home language is German. This also applies to girls who do not speak German at home. The finding that children and adolescents who speak one or more languages in addition to German at home are more frequently affected by overweight is evident in both age groups (Figure 1 and Annex Table 2). In terms of SES, children and adolescents from the high SES group who do not speak German at home are more frequently affected by overweight than children and adolescents who speak only German at home. The same applies to those who speak German at home in addition to one or more further languages (Figure 1 and Annex Table 2).

##### Alcohol consumption

**Figure 2:**
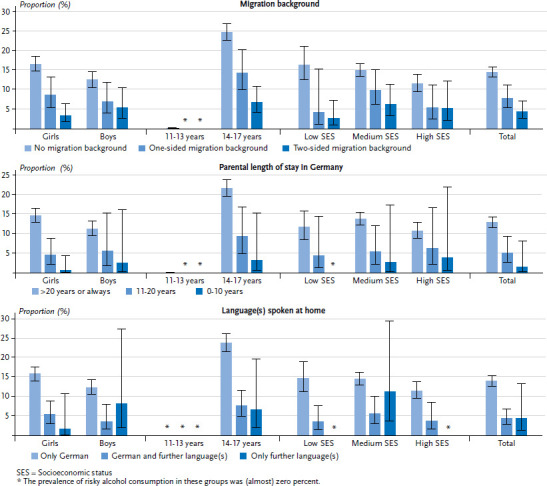
Prevalence of risky alcohol consumption among 11- to 17-year-olds by sociodemographic and migration-related characteristics (n=3,098 girls, n=2,797 boys) Source: KiGGS Wave 2 (2014-2017)

As much as 14.5%, children and adolescents without a migration background state that they consume risky amounts of alcohol significantly more often than those with a one-sided or two-sided migration background (Figure 2 and Annex Table 3). This finding holds true for both sexes. Among 14- to 17-year-olds, young people with a one- or two-sided migration background consume risky amounts of alcohol significantly less frequently than those without a migration background. Differences were also identified between the SES groups with regard to migration background. Whereas in the low and high SES groups children and adolescents with a one-sided migration background consume risky amounts of alcohol less frequently, in the low and medium SES group this applies to those with a two-sided migration background (Figure 2 and Annex Table 3). Children and adolescents whose parents have lived in Germany for more than 20 years or for their entire life have a higher frequency of risky alcohol consumption than those whose parents have lived in Germany for less than 20 years (Annex Table 3). The subgroup analysis demonstrates that the same applies to girls and to 14- to 17-year-olds. In the group with a medium SES, those whose parents have been living in Germany for more than 20 years or for their entire life consume risky amounts of alcohol significantly more frequently than children and adolescents whose parents have lived in Germany between 11 and 20 years (Figure 2 and Annex Table 3).

Differences in the frequency of risky alcohol consumption were also associated with the language spoken at home. Children and adolescents whose only home language is German state that they consume risky amounts of alcohol about three times more frequently (14.0%) than those who speak one or more further languages or who do not speak German at home (Figure 2 and Annex Table 3). Among the boys in all three SES groups, those who speak German at home alongside one or more further languages have a lower frequency of alcohol consumption than those whose only home language is German. In addition, among the girls and 14- to 17-year-olds, those who do not speak German at home consume risky amounts of alcohol less frequently than their peers (Figure 2 and Annex Table 3).

##### Utilisation of outpatient medical care

In total, more than three quarters of children and adolescents have made use of outpatient paediatric treatment or general medical services over the last twelve months. No migration-related differences were identified in the subgroups by sex, age, SES or parental length of stay (Annex Table 4).

However, one difference was found according to the language spoken at home in general and in one subgroup: children and adolescents in the high SES group who speak one or more further languages in addition to German at home make use of paediatric treatment and/or general medical services more frequently than those who speak only German at home (Annex Table 4).

## 4. Discussion

This article aims to describe the health situation of 11- to 17-year-olds based on the core indicators of the Federal Health Reporting on people with a migration background from the fields of health status, health behaviour and health care. In order to account for the diversity found among children and adolescents, the data were also stratified by additional migration-related and sociodemographic characteristics that go beyond the concept of migration background.

Self-assessments of general health (subjective health) were used to provide information about health status. Various longitudinal studies have demonstrated that self-assessments are useful predictors for the utilisation of medical services, chronic diseases, and mortality [29]. Most children and adolescents rated their general health as very good or good. Only few differences according to migration-related characteristics in individual subgroups were identified.

With regard to the prevalence of overweight (including obesity) the analyses of KiGGS Wave 2 indicate migration-related differences between children and adolescents. Hence, 11- to 17-year-olds with a migration background are more frequently affected by overweight than their peers without a migration background. According to some explanative approaches, the higher prevalence of overweight can be attributed, among other things, to the partially unhealthier diets among children and adolescents with a migration background [30]. The results of the KiGGS baseline study show that both a longer length of stay in Germany and belonging to the group of second-generation migrants are associated with higher levels of consumption of soft drinks and fast foods etc. In addition, children and adolescents with a two-sided migration background, especially girls, tend to have a higher prevalence of physical inactivity than their peers without a migration background [14].

However, both risk factors for overweight – an unhealthy diet and lower levels of physical activity – cannot be attributed to a migration background alone; therefore, a more in-depth analysis is required of the causes of overweight. It is possible that the financial restrictions that migrant families may face lead to a lower consumption of healthy foods and less use of leisure activities involving physical activity [21]. Living environments provide a further possible explanation and, thus, the availability of fast food restaurants and (the lack of) local leisure activities [31, 32]. This association could be analysed in the future by linking survey data with geodata [33]. However, as a limiting factor it should be noted that self-reported data on overweight were used from the KiGGS study, as these are available for all participants. Body weight and height were measured in a standardised manner in the examination part of the KiGGS survey, however, this was not offered to all participating children and adolescents. Compared to self-reported data, examination data are regarded as more reliable indicators. However, the number of cases is too low for differentiated analyses within the group of children and adolescents with a migration background in the age group under consideration. In contrast, the analyses show that children and adolescents with a one- or two-sided migration background consume risky amounts of alcohol significantly less frequently than their peers without a migration background. Previous research suggests associations between alcohol consumption and the presence of a migration background for e.g. cultural or religious reasons depending on the country of birth [14, 34, 35]. Due to the small number of cases and the lack of relevant determinants in the KiGGS survey (such as religious affiliation), this association cannot be further investigated in this article. No migration-related differences were identified among children and adolescents in terms of the utilisation of health care services: the majority of children and adolescents had used paediatric and/or general medical services during the last twelve months independent of an own or familial history of migration. Children and adolescents with a migration background only showed slight differences in the utilisation of outpatient medical care with regard to parental length of stay. Thus, a shorter length of parental stay in Germany was associated with a slightly lower utilisation of paediatric and general medical services. This may be due to the lack of knowledge about the German health system that migrants may have resulting from the shorter length of stay. In addition, experiences with discrimination and structural barriers related, i.a., to residence status can provide further explanations [36, 37]. It is surprising, however, that language, which the current literature identifies as a barrier to accessing health care, appears to have no association with the utilisation of paediatric or general health care services. However, it is important to note as another limiting factor of the KiGGS study, that the language spoken at home only provides an approximation of a person’s German language skills. Moreover, KiGGS Wave 2 did not collect data on ‘self-assessed German language proficiency’ as recommended in the migration-related indicator set of the Federal Health Reporting.

Overall, the results show that children and adolescents with and without a migration background have a similar level of good health. However, there are differences in outcomes associated with health behaviour (diet and physical activity, substance use). Although the KiGGS Wave 2 sample almost reflects the actual population with regard to the proportion of families with a migration background, an in-depth analysis of the differences in health behaviour would require larger case numbers in order to account for the diversity found among children and adolescents with a migration background. At the same time, the current analyses merely represent initial results. As a next step, multivariate models are required to examine the impact of migration-related variables after controlling for sociodemographic and socioeconomic characteristics. For example, in order to assess the validity of the assumption that an unhealthy diet and a lack of physical activity are primarily associated with financial constraints rather than a migration background, families with a low SES – with and without a migration background – should be more strongly integrated in future health surveys. Irrespective of their migration background, however, it is generally difficult to win people from this status group for surveys [15, 38].

Another problem concerning case numbers arises when comparing among children and adolescents with migration background: Differences in terms of utilisation of health care services may exist between those with migration background and those without German citizenship depending on their residency status (temporary versus permanent residence permits) and associated legal access to healthcare. However, these differences cannot be investigated using current data due to the small number of cases that are available.

In addition to the number of cases, the insufficient consideration of migration-sensitive concepts in KiGGS Wave 2 represents a further limitation. For example, data on religious affiliation could help explain the lower level of alcohol consumption among children and adolescents with a migration background – this indicator cannot be substituted by ‘origin from a country with a predominantly Muslim population’ just as the indicator ‘language spoken at home’ cannot adequately replace the self-assessed German language proficiency.

As part of the framework provided by the Robert Koch Institute’s Improving Health Monitoring in Migrant Populations (IMIRA) project, various concepts that have been used within the health monitoring in the past have been revised and expanded for migration-sensitive components (see also Concepts for migration-sensitive health monitoring in this issue of the Journal of Health Monitoring). In the future, these concepts are to be used to account for the diversity of the population in Germany and to assess approaches that go beyond the mere concept of migration background when studying health differences. It is essential to ensure the long-term, representative involvement of all population groups in health monitoring at the Robert Koch Institute. In order to ensure that people with a migration background are properly integrated into health surveys, the IMIRA project has further developed the migrationsensitive methods of participant recruitment and survey methods used in the KiGGS baseline study and in KiGGS Wave 2 [39]. In the future, the results will be incorporated into an integrated overall concept of health monitoring at the Robert Koch Institute.

Despite the migration-related limitations outlined above, the KiGGS study constitutes a unique data source to describe the health of children and adolescents in Germany. In particular, the KiGGS baseline study and KiGGS Wave 2, which were conceived as interview and examination surveys, are unique as they made use of migration-sensitive study designs. Another peculiarity is the KiGGS cohort, which allows life course analyses. With this approach, long-term effects of exposures in different phases of life and environments can be analysed and correlated [5, 40]. Life course epidemiology has not yet been widely applied to people with migration background due to the lack of data. However, with the KiGGS study, data are now available to analyse the complex conditional factors that affect the health of children and adolescents with a migration background.

## Key statements

- The data from KiGGS Wave 2 provides the opportunity for a differentiated description of the health of children and adolescents based on migration-related and sociodemographic characteristics.
- No differences in self-assessed general health were identified between 11- to 17-year-olds with and without a migration background.
- Children and adolescents with a migration background are more frequently overweight than their peers without a migration background but consume risky amounts of alcohol less frequently.
- The utilisation of paediatric or general medical services did not differ between children and adolescents with and without a migration background.
- For selected health indicators, there are significant differences in sociodemographic and migration-related characteristics between 11- to 17-year-olds.

## Appendix

**Annex Table 1:** Prevalence of self-assessed very good or good general health among 11- to 17-year-olds by sociodemographic and migration-related characteristics (n=3,156 girls, n=2,874 boys) Source: KiGGS Wave 2 (2014-2017)

		n	%	(95% CI)	n	%	(95% CI)	OR	p-value	n	%	(95% CI)	OR	p-value
**Migration background**		**None (Ref.)**			**One-sided**					**Two-sided**				
**Sex**	Girls	2,552	87.7	(86.0-89.2)	274	86.6	(80.4-91.1)	0.91	0.690	330	83.8	(77.8-88.5)	0.73	0.150
	Boys	2,385	90.9	(89.3-92.3)	211	91.9	(84.4-96.0)	1.14	0.739	278	90.4	(85.5-93.8)	0.94	0.811
**Age group**	11-13 years	2,262	92.1	(90.4-93.5)	241	90.7	(83.3-95.1)	0.84	0.637	261	91.9	(87.6-94.7)	0.97	0.907
	14-17 years	2,675	87.5	(85.7-89.1)	244	87.7	(81.8-91.9)	1.02	0.919	347	84.6	(79.5-88.6)	0.79	0.254
**SES**	Low	505	86.1	(81.4-89.7)	64	83.1	(67.2-92.2)	0.79	0.627	197	85.1	(78.7-89.8)	0.92	0.783
	Medium	3,227	89.4	(87.9-90.8)	283	89.7	(84.9-93.1)	1.03	0.901	327	89.5	(84.3-93.1)	1.01	0.972
	High	1,193	91.9	(90.0-93.4)	135	93.0	(86.8-96.4)	1.18	0.661	78	88.6	(77.5-94.6)	0.69	0.391
**Total**		4,937	89.4	(88.1-90.5)	485	89.1	(84.8-92.3)	0.97	0.892	608	87.1	(83.6-90.0)	0.80	0.206
**Parental length of stay in Germany**		**> 20 years or entire life (Ref.)**			**11-20 years**					**0-10 years**				
**Sex**	Girls	2,734	88.4	(86.8-89.9)	172	77.5	**(68.6-84.5)**	**0.45**	**0.002**	58	88.1	(66.3-96.5)	0.97	0.963
	Boys	2,549	91.5	(90.0-92.7)	134	87.7	(76.6-93.9)	0.66	0.322	52	93.1	(83.0-97.4)	1.25	0.668
**Age group**	11-13 years	2,402	92.8	(91.2-94.1)	168	87.2	(78.9-92.6)	0.53	0.054	57	92.4	(80.8-97.2)	0.95	0.926
	14-17 years	2,881	88.2	(86.6-89.6)	138	78.3	**(68.9-85.5)**	**0.48**	**0.007**	53	89.1	(68.2-96.9)	1.10	0.893
**SES**	Low	554	87.4	(83.1-90.8)	95	76.9	**(65.1-85.6)**	**0.48**	**0.044**	33	92.9	(80.2-97.7)	1.89	0.311
	Medium	3,414	90.2	(88.9-91.4)	156	87.5	(80.6-92.1)	0.76	0.308	59	86.8	(65.2-95.8)	0.71	0.597
	High	1,303	91.7	(89.9-93.2)	53	89.6	(78.8-95.2)	0.78	0.571	17	100.0	-	-	-
**Total**		5,283	90.0	(88.9-91.0)	306	82.4	**(75.6-87.6)**	**0.52**	**0.004**	110	90.7	(79.7-96.0)	1.08	0.869
**Language(s) spoken at home**		**Only German (Ref.)**			**German and further language(s)**					**Only further language(s)**				
**Sex**	Girls	2,672	87.5	(85.8-89.0)	383	84.8	(79.5-89.0)	0.80	0.270	58	85.5	(67.4-94.4)	0.84	0.748
	Boys	2,476	91.4	(89.8-92.7)	320	88.2	(82.1-92.4)	0.70	0.199	40	98.9	**(92.8-99.6)**	**8.75**	**0.032**
**Age group**	11-13 years	2,362	91.8	(90.1-93.2)	325	91.5	(86.4-94.8)	0.97	0.908	42	97.1	(89.1-99.3)	2.97	0.132
	14-17 years	2,786	87.9	(86.2-89.4)	378	83.1	**(78.1-87.2)**	**0.68**	**0.043**	56	88.8	(72.4-96.0)	1.09	0.881
**SES**	Low	540	86.7	(82.1-90.2)	177	80.2	(72.1-86.4)	0.62	0.117	33	96.0	(77.9-99.4)	3.71	0.184
	Medium	3,345	89.5	(88.1-90.8)	387	90.6	(86.4-93.6)	1.12	0.612	48	86.4	(61.1-96.2)	0.74	0.678
	High	1,251	91.7	(89.9-93.2)	132	93.0	(86.2-96.6)	1.21	0.638	16	82.9	(47.2-96.3)	0.44	0.342
**Total**		5,148	89.6	(88.3-90.6)	703	86.5	(82.6-89.7)	0.75	0.105	98	91.3	(79.7-96.6)	1.24	0.678

Bold = statistically significant in comparison to the reference group, CI = Confidence interval, OR = Odds ratio, Ref. = Reference group, SES = Socioeconomic status

**Annex Table 2:** Prevalence of overweight (including obesity) among 11- to 17-year-olds by sociodemographic and migration-related characteristics (n=3,010 girls, n=2,751 boys) Source: KiGGS Wave 2 (2014-2017)

		n	%	(95% CI)	n	%	(95% CI)	OR	p-value	n	%	(95% CI)	OR	p-value
**Migration background**		**None (Ref.)**			**One-sided**					**Two-sided**				
**Sex**	Girls	2,447	10.0	(8.4-11.8)	254	16.0	**(11.5-21.7)**	**1.71**	**0.015**	309	19.2	**(13.6-26.4)**	**2.14**	**0.002**
	Boys	2,292	12.4	(10.7-14.4)	197	22.4	**(14.8-32.3)**	**2.04**	**0.011**	262	20.5	**(15.1-27.1)**	**1.82**	**0.006**
**Age group**	11-13 years	2,163	9.6	(8.0-11.4)	223	19.3	**(13.2-27.5)**	**2.26**	**0.001**	239	13.9	(9.7-19.6)	1.52	0.059
	14-17 years	2,576	12.4	(10.8-14.2)	228	18.6	(12.5-26.6)	1.62	0.054	332	22.8	**(17.5-29.0)**	**2.09**	**< 0.001**
**SES**	Low	479	17.5	(14.0-21.6)	63	31.6	**(19.7-46.5)**	**2.18**	**0.031**	181	25.1	(18.1-33.8)	1.58	0.065
	Medium	3,097	11.8	(10.3-13.6)	262	17.6	**(12.3-24.4)**	**1.59**	**0.042**	308	15.1	(10.7-20.9)	1.32	0.222
	High	1,151	4.7	(3.5-6.3)	124	10.3	(5.0-20.1)	2.33	0.059	77	12.9	**(5.8-26.4)**	**2.99**	**0.029**
**Total**		4,739	11.2	(10.1-12.5)	451	18.9	**(14.2-24.7)**	**1.85**	**0.001**	571	19.8	**(15.9-24.4)**	**1.95**	**< 0.001**
**Parental length of stay in Germany**		**> 20 years or entire life (Ref.)**			**11-20 years**					**0-10 years**				
**Sex**	Girls	2,617	10.8	(9.1-12.7)	162	19.0	**(12.4-28.1)**	**1.94**	**0.020**	54	16.8	(6.8-35.9)	1.67	0.328
	Boys	2,452	13.9	(12.1-15.8)	125	24.9	**(16.1-36.4)**	**2.06**	**0.018**	48	10.3	(4.2-23.2)	0.72	0.496
**Age group**	11-13 years	2,298	9.6	(8.1-11.3)	153	22.6	**(15.4-31.9)**	**2.75**	**< 0.001**	51	13.6	(6.5-26.5)	1.49	0.340
	14-17 years	2,771	14.2	(12.6-16.1)	134	21.2	(13.7-31.2)	1.62	0.096	51	13.3	(4.7-32.4)	0.92	0.890
**SES**	Low	526	23.1	(18.6-28.5)	90	21.2	(13.0-32.7)	0.89	0.733	30	17.2	(6.1-39.9)	0.69	0.540
	Medium	3,274	11.9	(10.4-13.6)	147	23.8	**(15.9-34.1)**	**2.32**	**0.002**	55	11.2	(5.2-22.5)	0.94	0.877
	High	1,257	5.4	(4.2-6.9)	48	17.0	**(6.0-39.9)**	**3.62**	**0.038**	17	5.9	(0.9-31.2)	1.11	0.916
**Total**		5,069	12.4	(11.2-13.7)	287	21.8	**(16.2-28.7)**	**1.97**	**0.001**	102	13.4	(6.9-24.5)	1.10	0.803
**Language(s) spoken at home**		**Only German (Ref.)**			**German and further language(s)**					**Only further language(s)**				
**Sex**	Girls	2,560	10.4	(8.8-12.1)	358	17.2	**(12.2-23.5)**	**1.79**	**0.012**	53	23.4	**(12.1-40.5)**	**2.65**	**0.021**
	Boys	2,380	12.9	(11.2-14.8)	300	21.6	**(16.2-28.2)**	**1.86**	**0.004**	36	21.8	(9.9-41.4)	1.88	0.187
**Age group**	11-13 years	2,257	9.8	(8.2-11.6)	299	16.7	**(11.8-23.1)**	**1.85**	**0.007**	37	19.5	(8.9-37.7)	2.24	0.094
	14-17 years	2,683	13.0	(11.4-14.7)	359	21.2	**(15.8-27.7)**	**1.80**	**0.005**	52	23.9	(13.0-39.9)	2.11	0.057
**SES**	Low	514	19.7	(16.1-23.9)	164	24.9	(17.8-33.8)	1.35	0.243	30	24.2	(11.0-45.2)	1.30	0.593
	Medium	3,212	11.9	(10.5-13.6)	360	16.4	(11.9-22.1)	1.45	0.070	44	19.7	(9.3-37.0)	1.81	0.191
	High	1,203	4.9	(3.7-6.5)	127	11.3	**(5.6-21.6)**	**2.48**	**0.042**	15	28.1	**(9.9-58.2)**	**7.59**	**0.003**
**Total**		4,940	11.7	(10.5-12.9)	658	19.4	**(15.5-24.0)**	**1.83**	**< 0.001**	89	22.7	**(13.9-34.8)**	**2.23**	**0.010**

Bold = statistically significant in comparison to the reference group, CI = Confidence interval, OR = Odds ratio, Ref. = Reference group, SES = Socioeconomic status

**Annex Table 3:** Prevalence of risky alcohol consumption (AUDIT-C) among 11- to 17-year-olds by sociodemographic and migration-related characteristics (n=3,098 girls, n=2,797 boys) Source: KiGGS Wave 2 (2014-2017)

		n	%	(95% CI)	n	%	(95% CI)	OR	p-value	n	%	(95% CI)	OR	p-value
**Migration background**		**None (Ref.)**			**One-sided**					**Two-sided**				
**Sex**	Girls	2,504	16.6	(14.8-18.6)	267	8.6	**(5.4-13.2)**	**0.47**	**0.004**	327	3.3	**(1.7-6.4)**	**0.17**	**< 0.001**
	Boys	2,311	12.5	(10.6-14.6)	207	7.0	**(4.0-11.9)**	**0.52**	**0.037**	279	5.4	**(2.7-10.5)**	**0.40**	**0.016**
**Age group**	11-13 years	2,219	0.1	(0.0-0.3)	236	0.0	-	-	-	266	0.0	-	-	-
	14-17 years	2,596	24.7	(22.6-27.0)	238	14.3	**(10.0-20.2)**	**0.51**	**0.002**	340	6.8	**(4.2-10.8)**	**0.22**	**< 0.001**
**SES**	Low	487	16.4	(12.6-21.2)	63	4.2	**(1.0-15.3)**	**0.22**	**0.038**	197	2.6	**(0.9-7.2)**	**0.14**	**0.001**
	Medium	3,158	15.0	(13.4-16.7)	277	9.9	(6.4-15.1)	0.63	0.058	325	6.2	**(3.3-11.3)**	**0.38**	**0.006**
	High	1,159	11.6	(9.5-14.0)	131	5.4	**(2.5-11.2)**	**0.44**	**0.045**	78	5.2	(2.1-12.2)	0.42	0.071
**Total**		4,815	14.5	(13.2-15.9)	474	7.8	**(5.4-11.2)**	**0.50**	**0.001**	606	4.4	**(2.7-7.1)**	**0.27**	**< 0.001**
**Parental length of stay in Germany**		**> 20 years or entire life (Ref.)**			**11-20 years**					**0-10 years**				
**Sex**	Girls	2,685	14.7	(13.0-16.6)	171	4.6	**(2.3-8.9)**	**0.28**	**< 0.001**	56	0.6	**(0.1-4.4)**	**0.04**	**0.001**
	Boys	2,472	11.4	(9.6-13.4)	137	5.6	(1.9-15.4)	0.46	0.179	52	2.5	(0.3-16.3)	0.20	0.122
**Age group**	11-13 years	2,359	0.1	(0.02-0.3)	170	0.0	-	-	-	57	0.0	-	-	-
	14-17 years	2,798	21.8	(19.7-24.0)	138	9.4	**(5.0-17.1)**	**0.37**	**0.005**	51	3.2	**(0.6-15.5)**	**0.12**	**0.016**
**SES**	Low	535	11.8	(8.6-16.0)	97	4.5	(1.3-14.6)	0.35	0.128	32	0.0	-	-	-
	Medium	3,342	14.0	(12.4-15.6)	158	5.5	**(2.3-12.2)**	**0.36**	**0.024**	59	2.8	(0.4-17.6)	0.18	0.092
	High	1,268	10.8	(8.9-13.1)	51	6.4	(2.2-16.9)	0.56	0.307	16	3.9	(0.6-22.2)	0.34	0.272
**Total**		5,157	13.0	(11.7-14.4)	308	5.1	**(2.7-9.5)**	**0.36**	**0.003**	108	1.6	**(0.3-8.2)**	**0.11**	**0.011**
**Language(s) spoken at home**		**Only German (Ref.)**			**German and further language(s)**					**Only further language(s)**				
**Sex**	Girls	2,622	15.8	(14.0-17.6)	380	5.4	**(3.2-8.9)**	**0.31**	**< 0.001**	55	1.6	**(0.2-10.8)**	**0.09**	**0.017**
	Boys	2,403	12.3	(10.5-14.4)	317	3.6	**(1.6-8.0)**	**0.26**	**0.002**	40	8.1	(2.0-27.4)	0.63	0.539
**Age group**	11-13 years	2,317	0.1	(0.0-0.3)	0	0.0	–	–	–	42	0.0	–	–	–
	14-17 years	2,708	23.8	(21.6-26.1)	328	7.6	**(4.9-11.6)**	**0.26**	**< 0.001**	53	6.7	**(2.0-19.7)**	**0.23**	**0.021**
**SES**	Low	521	14.7	(11.2-19.0)	179	3.5	**(1.5-7.7)**	**0.21**	**< 0.001**	31	0.0	–	–	–
	Medium	3,276	14.6	(13.0-16.3)	382	5.6	**(3.1-10.0)**	**0.35**	**0.002**	48	11.2	(3.7-29.5)	0.74	0.625
	High	1,217	11.5	(9.6-13.9)	129	3.8	**(1.7-8.5)**	**0.30**	**0.006**	15	0.0	–	–	–
**Total**		5,025	14.0	(12.7-15.4)	697	4.5	**(2.9-6.9)**	**0.29**	**< 0.001**	95	4.5	**(1.4-13.4)**	**0.29**	**0.044**

Bold = statistically significant in comparison to the reference group, CI = Confidence interval, OR = Odds ratio, Ref. = Reference group, SES = Socioeconomic status, AUDIT-C = Alcohol Use Disorder Identification Test (-Consumption)

**Annex Table 4:** 12-month prevalence of the utilisation of outpatient paediatric and/or general medical services among 11- to 17-year-olds by sociodemographic and migration-related characteristics (n=3,139 girls, n=2,810 boys) Source: KiGGS Wave 2 (2014-2017)

		n	%	(95% CI)	n	%	(95% CI)	OR	p-value	n	%	(95% CI)	OR	p-value
**Migration background**		**None (Ref.)**			**One-sided**					**Two-sided**				
**Sex**	Girls	2,547	78.9	(76.7-81.0)	268	84.1	(77.5-89.1)	1.41	0.127	324	80.3	(73.4-85.8)	1.09	0.699
	Boys	2,335	77.6	(75.2-80.0)	207	74.1	(66.0-80.8)	0.82	0.336	268	74.0	(66.7-80.2)	0.82	0.324
**Age group**	11-13 years	2,267	81.3	(78.7-83.7)	239	85.0	(79.0-89.5)	1.30	0.229	256	81.3	(73.3-87.3)	1.00	0.997
	14-17 years	2,615	76.1	(73.7-78.3)	236	74.5	(67.5-80.4)	0.92	0.630	336	75.1	(68.5-80.7)	0.95	0.776
**SES**	Low	496	80.4	(74.9-85.0)	66	88.7	(78.2-94.5)	1.92	0.123	191	79.8	(72.0-85.8)	0.96	0.877
	Medium	2,566	78.9	(76.9-80.9)	273	75.7	(69.8-80.8)	0.83	0.238	321	74.2	(66.7-80.5)	0.77	0.186
	High	904	74.4	(70.8-77.7)	133	80.2	(70.0-87.5)	1.39	0.258	76	81.8	(71.9-88.7)	1.54	0.157
**Total**		4,882	78.3	(76.5-79.9)	475	79.4	(75.0-83.2)	1.07	0.623	592	77.2	(72.4-81.4)	0.94	0.687
**Parental length of stay in Germany**		**> 20 years or entire life (Ref.)**			**11-20 years**					**0-10 years**				
**Sex**	Girls	2,724	79.6	(77.5-81.5)	167	84.5	(76.7-90.1)	1.40	0.209	57	63.7	**(44.8-79.1)**	**0.45**	**0.049**
	Boys	2,494	76.8	(74.5-78.9)	131	75.7	(65.6-83.6)	0.94	0.824	49	79.3	(61.5-90.2)	1.16	0.752
**Age group**	11-13 years	2,407	81.5	(79.0-83.7)	164	82.8	(74.5-88.8)	1.09	0.744	54	78.6	(60.3-89.9)	0.84	0.705
	14-17 years	2,811	75.9	(73.7-77.9)	134	78.2	(68.7-85.4)	1.14	0.607	52	65.5	(47.4-80.0)	0.61	0.199
**SES**	Low	546	81.2	(75.6-85.8)	92	81.7	(70.3-89.4)	1.03	0.927	31	76.5	(51.7-90.8)	0.75	0.627
	Medium	3,376	78.2	(76.2-80.2)	154	78.8	(70.2-85.4)	1.03	0.891	57	66.2	(49.0-79.9)	0.54	0.102
	High	1,285	75.3	(71.9-78.4)	52	79.7	(66.5-88.6)	1.29	0.480	17	81.9	(57.0-93.9)	1.49	0.526
**Total**		5,218	78.1	(76.5-79.7)	298	80.3	(74.2-85.2)	1.14	0.495	106	71.6	(58.3-81.9)	0.70	0.259
**Language(s) spoken at home**		**Only German (Ref.)**			**German and other language(s)**					**Only other language(s)**				
**Sex**	Girls	2,664	79.2	(77.0-81.2)	375	82.7	(77.6-86.9)	1.26	0.204	57	74.6	(56.5-86.9)	0.77	0.545
	Boys	2,420	77.0	(74.6-79.3)	312	75.5	(68.8-81.2)	0.92	0.651	40	77.2	(59.7-88.5)	1.01	0.989
**Age group**	11-13 years	2,366	81.4	(78.9-83.6)	320	83.2	(76.5-88.2)	1.13	0.600	41	80.7	(62.6-91.3)	0.96	0.923
	14-17 years	2,718	75.8	(73.4-78.0)	367	76.4	(70.5-81.4)	1.04	0.833	56	73.5	(58.5-84.5)	0.89	0.743
**SES**	Low	532	80.1	(74.5-84.7)	171	83.3	(75.8-88.9)	1.24	0.445	33	76.8	(56.6-89.4)	0.82	0.698
	Medium	3,307	78.5	(76.5-80.4)	381	74.6	(68.4-79.9)	0.80	0.194	47	79.6	(66.3-88.6)	1.07	0.854
	High	1,234	75.1	(71.6-78.3)	130	84.3	**(77.4-93.3)**	**1.77**	**0.016**	16	55.8	(34.5-75.1)	0.42	0.058
**Total**		5,084	78.1	(76.4-79.7)	687	79.1	(75.0-82.7)	1.06	0.654	97	75.7	(63.9-84.6)	0.87	0.650

Bold = statistically significant in comparison to the reference group, CI = Confidence interval, OR = Odds ratio, Ref. = Reference group, SES = Socioeconomic status
